# Bottlenecks and predictors of coverage and adherence outcomes for a micronutrient powder program in Ethiopia

**DOI:** 10.1111/mcn.12807

**Published:** 2019-10-17

**Authors:** Alison Tumilowicz, Jean‐Pierre Habicht, Mduduzi N.N. Mbuya, Ty Beal, Robert Ntozini, Fabian Rohner, Gretel H. Pelto, Tezera Fisseha, Jemal Haidar, Nigussie Assefa, Hana Yemane Wodajo, Telahun Teka Wolde, Lynnette M. Neufeld

**Affiliations:** ^1^ Global Alliance for Improved Nutrition Geneva Switzerland; ^2^ Division of Nutritional Sciences Cornell University Ithaca New York USA; ^3^ Global Alliance for Improved Nutrition Washington DC USA; ^4^ Department of Environmental Science and Policy University of California Davis California USA; ^5^ Zvitambo Institute for Maternal and Child Health Research Harare Zimbabwe; ^6^ GroundWork Fläsch Switzerland; ^7^ Institute for Education, Health and Development Addis Ababa Ethiopia; ^8^ School of Public Health, College of Health Sciences Addis Ababa University Addis Ababa Ethiopia; ^9^ Health Services Quality Directorate Federal Ministry of Health Addis Ababa Ethiopia

## Abstract

A theory‐driven evaluation was conducted to assess performance of a trial to deliver micronutrient powder (MNP) through the Ethiopian Ministry of Health. We adapted an approach to coverage assessment, originally developed to identify bottlenecks in health service delivery, to examine sequential program outcomes and their correlates using cross‐sectional survey data of caregivers of children 6–23 months (*N* = 1915). Separate multivariable Poisson regression models were used to estimate adjusted risk ratios of conceptually relevant determinants of coverage and adherence. Caregivers of children >11 months were more likely to have received MNP than caregivers of younger infants, yet children 12–17 months were 32% (*P* < 0.001) and children 18–23 months 38% (*P* < 0.001) less likely to have been fed MNP in the 14 days preceding the survey than children 6–11 months. Among caregivers who initiated feeding MNP, the most frequently reported reasons for discontinuing use were not obtaining additional supply (36.1%) and perceived child rejection of food with MNP (22.9%). For each additional time a caregiver met with frontline workers in the 3 months preceding the survey, they were 13% more likely to have recently fed MNP (*P* < 0.001). Caregivers' perception that MNP produced positive changes in children was associated with a 14% increase in the likelihood of having recently fed it (*P* < 0.001). These results emphasize the importance of counselling for MNP and infant and young child feeding for initial use and the importance of multiple contacts with frontline workers for continued use.

**Key messages**

- Continued MNP use was positively associated with living in rural Tigray Region, child age <12 months, female child, increased frequency of contact with frontline workers and perceived positive outcomes from feeding MNP.
- Caregivers face challenges to continue feeding MNP through their children's second year of life as they experience periods of illness and poor appetite, progress through developmental stages that affect feeding behaviours and stop routine attendance at health services.
- Frequent contacts with frontline workers foster continued use of MNP, likely through ensuring caregivers have supply of MNP, alerting caretakers to positive changes and potential negative side effects in the child, and helping address MNP and IYC feeding difficulties.

## 1 INTRODUCTION

Point‐of‐use fortification of complementary foods with iron‐containing micronutrient powders (MNP) is recommended by the World Health Organization (WHO) as a strategy to improve iron status and reduce anaemia in infants and young children (World Health Organization, 2016). Although MNP interventions have been implemented across 50 countries (United Nations International Children's Emergency Fund, 2015), achieving high program coverage and adherence to MNP recommendations is an ongoing challenge (Reerink et al., 2017). The biological pathway to impact for MNP is well articulated (De‐Regil, Suchdev, Vist, Walleser, & Peña‐Rosas, 2013), but program implementers are often constrained by insufficient understanding of the factors and processes underlying successful delivery of the intervention (Vossenaar et al., 2017).

Only 7% of Ethiopian infants and young children 6–23 months of age consume the minimum number of food groups associated with better nutrient adequacy and growth (Arimond & Ruel, 2004; Central Statistical Agency Ethiopia & ICF International, 2007; Marriott, White, Hadden, Davies, & Wallingford, 2012). A recent national micronutrient survey found that 34% of children under 5 years of age experience anaemia, 18% are iron deficient, 14% vitamin A deficient and 35% zinc deficient (Ethiopian Public Health Institute, 2016). Point‐of‐use fortification of complementary foods with MNP has been demonstrated to improve growth and haemoglobin status among Ethiopian children 6–23 months of age (Samuel et al., 2018).

The Global Alliance for Improved Nutrition with funding from the Ministry of Foreign Affairs of the Netherlands and in partnership with the Ethiopian Ministry of Health (MOH) and Concern Worldwide pilot‐tested the delivery of MNP, locally branded as “Desta” (the Amharic and https://en.wikipedia.org/wiki/Tigrinya_language word for “joy, happiness”; produced by Hexagon, India), through the MOH's Health Extension Program. The MOH Health Extension Program deploys government‐salaried female health extension workers (HEW) to provide primary health care services in rural communities (Federal Ministry of Health: Health Extension and Education Center, 2006, 2007). From May 2016 to May 2017 in 11 *woredas* (geographic units equivalent to districts) of Amhara and Tigray Regions in Ethiopia, HEW and health centre staff integrated the delivery of MNP sachets and recommendations in their routine services, which include growth monitoring, cooking demonstrations, health and nutrition inter‐personal communication (e.g. counselling) and immunizations.

A theory‐driven, mixed‐method, formative process evaluation was conducted to elucidate how the intervention worked, with the goal of determining what aspects of program delivery and adherence went well, identifying where problems occurred and understanding the magnitude and causes of problems. To illustrate how the delivery of the program would result in continuous and appropriate use of MNP, we developed a program impact pathway (PIP; Habicht & Pelto, 2012) through consultations with program staff and an extensive literature review of factors associated with MNP adherence (Tumilowicz, Schnefke, Neufeld, & Pelto, 2017). The PIP described two sequential systems: (1) the program delivery system, which includes the processes by which the intervention is delivered to the household, and (2) the household delivery system, which consists of all steps required for a child to consume a biologically impactful dose (Carroll et al., 2007).

**Figure 1:**
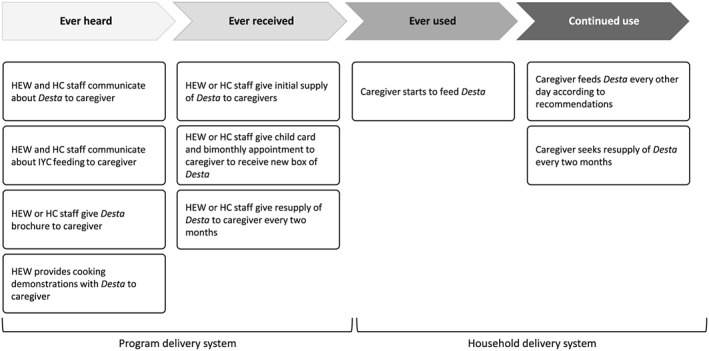
Program impact pathway for micronutrient powder delivery system in Ethiopia. HEW: health extension worker; HC: health centre staff; IYC: infant and young child; *Desta*, brand of micronutrient powder used in the trial

For MNP interventions, the processes underlying the household delivery system are based on adherence—the extent to which a caregiver's trial and adoption of behaviour and the child's consumption are congruent with the MNP recommendations offered by the delivery system (Tumilowicz et al., 2017; Vrijens et al., 2012). Figure 1 presents the flow of the PIP in the form of program component exposures and actions by the caregiver. The PIP describes the transmission of MNP and behaviour change communication (BCC) activities from the program delivery system to the household delivery system, as well as the sequence of activities to prepare food with MNP and feed it to the child.

The extent to which a nutrition program is delivered as intended substantially influences its potential for impact (Mbuya, Menon, Habicht, Pelto, & Ruel, 2013). This challenge is exacerbated in interventions and programs with either multiple or complex delivery systems (Pearce & Merletti, 2006). The key challenges in studying the handover processes within and between delivery systems are to conceptually and analytically elucidate them in order to ascertain their determinants and identify how to create conditions that improve the effectiveness of nutrition interventions (Mbuya et al., 2013). In this analysis, we adapt the Tanahashi approach to coverage assessment (Tanahashi, 1978), originally developed to identify bottlenecks in health service delivery, to describe changes in MNP coverage and adherence outcomes. We add to the Tanahashi method an examination of determinants for the sequential bottlenecks. Our intention is to contribute to the expanding evidence base on MNP programs and apply a novel analytic approach to examine the determinants of each outcome separately.

## 2 MATERIALS AND METHODS

### 2.1 MNP integration into MOH Health Extension Program

The trial aimed to deliver the MNP locally branded as *Desta* and infant and young child (IYC) feeding BCC activities to approximately 71,000 children 6–23 months in six *woredas* in Amhara Region (Kobo, Gubalafto, Delanta, Dessie Zuria, Kalu and Bati) and five *woredas* in Tigray Region (Saesi Tsaedaemba, Mereb Lehe, Werie Lehe, Gulomahda and Ahferom). The distribution of *Desta* was integrated into the IYC feeding BCC strategy developed by Alive & Thrive and implemented through the MOH's Health Extension Program (Kim et al., 2015, 2016). The MOH, Concern Worldwide and Global Alliance for Improved Nutrition created guidelines, a training manual, job aids and materials for caregivers, which included information on the composition and purpose of *Desta*, instruction on food preparation with *Desta* and contraindications and potential side effects of *Desta* (see Table SS1 in the supporting information for key messages regarding *Desta* that HEW and health centre workers were trained to provide to caregivers). Starting in May 2016 and until May 2017, HEW or health centre staff provided a box containing 30 *Desta* sachets every 2 months to caregivers at the health post, health centre or during home visits. Caregivers were instructed to feed food mixed with one sachet of *Desta* to the child 6–23 months of age every other day and return to the HEW or health centre for a new supply after 2 months. At the initial distribution, caregivers were provided with a “*Desta* child card” that could be used to keep track of distribution dates. The card included appointment dates for caregivers to return to receive a new box of *Desta*, which was scheduled 2 months after the date of the current distribution.

### 2.2 Study design and population

We identified two main questions to be addressed in the evaluation: (1) what proportion of caregivers received the intervention and adhered to *Desta* recommendations and (2) how did the delivery and utilization processes that characterized the trial affect program outcomes, including coverage and adherence to recommendations regarding *Desta*. Examination of these two questions required a complex study design and mixed methods. Therefore, we created two intersecting streams of inquiry: (1) an examination of sequential program outcomes and their correlates using cross‐sectional survey data and (2) a focused ethnographic study (FES) to investigate delivery experiences and *Desta* use from the perspectives of caregivers. We report on the results of the FES in a separate paper of this supplement (Pelto et al., 2019). This paper presents key findings from a population‐based cross‐sectional survey conducted at the same time as the FES, approximately 8 months after initiation of MNP distribution.

The survey was conducted in the Tigray and Amhara Regions of Ethiopia during January and February 2017. Tigray Region has a population of almost five million people, of which 20% are urban (Central Statistical Agency of Ethiopia, 2007). Average household size is 4.4 persons who are predominantly (96%) https://en.wikipedia.org/wiki/Tigrinya_language‐speaking Tigray ethnic group. Agriculture is the main source of livelihood. Amhara Region has a population of over 17 million people, of which 12% are urban (Central Statistical Agency of Ethiopia, 2007). Average household size is 4.3, and 92% are from the https://en.wikipedia.org/wiki/Semitic_languages‐speaking https://en.wikipedia.org/wiki/Amhara_people ethnic group. Agriculture is the main occupation.

### 2.3 Sampling

The implementation area was divided into three strata: rural Amhara, rural Tigray and urban Amhara and Tigray combined. As it was not budgetarily feasible to collect data for the number of enumeration areas (EA) required to create separate urban strata for the regions, urban areas in Amhara and Tigray were combined into a single stratum, which permitted comparisons across the two rural areas and the combined urban area. The sampling frame used for the first stage of sampling was provided by the Federal Central Statistical Agency of Ethiopia. In the first stage of sampling, 40 EA, which are the smallest census unit and contained about 200 households, were randomly selected in each stratum with the probability of selection for each EA being proportional to the number of households in that EA. Prior to conducting the survey, six of the urban EA were excluded from the survey because a recent administrative reorganization allocated these EA to areas outside the trial's implementation *woredas*.

Prior to the second stage of sampling, a household listing was carried out in each selected EA to identify all potentially eligible children 6–23 months of age and to update the population size estimate. The resulting child lists in each EA were used in the second stage of sampling, which consisted of a simple random selection of 18 children 6–23 months of age in each EA. If the caregiver provided written informed consent, a questionnaire was used to collect data on *Desta* coverage and adherence outcomes, caregiver exposure to the elements of the trial design and perception‐of‐use factors, variables that could have affected program delivery exposure or adherence outcomes and covariates such as demographic factors.

The questionnaire response rate of the 2,052 selected mothers was 95.6% overall (*N* = 1,963), 96.8% (*N* = 697 of 720) in Tigray, 94.4% (*N* = 680 of 720) in Amhara and 95.8% (*N* = 586 of 612) in the urban stratum. After further elimination of 47 cases with implausible responses (e.g. reporting to have fed *Desta* despite not having heard of it or received it), the total sample of caregivers used in the estimation of outcomes was *N* = 1916 (93%). The sample size for the multivariable analysis was 1915 because one caregiver's age was missing and therefore excluded from the analyses.

### 2.4 Outcome variables

We adapted the Tanahashi (1978) approach to health program coverage assessment (Tanahashi, 1978), to the PIP of the Ethiopia trial and indicators collected by the survey. We specified four main sequential outcomes to analyse bottlenecks. The first two outcomes relate to the trial's program coverage and latter two to the household adherence to *Desta* recommendations:

- “Ever heard,” the proportion of all caregivers who ever heard of *Desta*; analogous to “availability coverage” as defined by Tanahashi
- “Ever received,” the proportion of all caregivers who ever received a box of *Desta*; analogous to “accessibility coverage” as defined by Tanahashi
- “Ever fed,” the proportion of all caregivers who ever fed *Desta* to the child; analogous to “contact coverage” as defined by Tanahashi
- “Recently fed,” the proportion of all caregivers who fed *Desta* to the child at least one time in the 14 days preceding the survey; analogous to “effectiveness coverage” as defined by Tanahashi

The data did not permit construction of a more accurate indicator of effectiveness coverage or adherence to recommendations (e.g. number of sachets used over the last 7 days). Although imperfect, we considered “used at least once within the 14 days preceding the survey” to be indicative of continued use and effectiveness coverage. Each outcome is conditional on having accomplished the previous one; for example, mothers can only have “ever fed” if they “ever received” *Desta*. For “ever heard,” the study population provides the comparator. A large difference in the proportions of the target population accomplishing adjacent outcomes implies the existence of a bottleneck in the PIP. The magnitude of the bottleneck is the attrition in proportions between one outcome and the next.

To examine the determinants of each step through which *Desta* is transferred from program implementers to caregivers and ultimately to their children, we computed the following outcomes' proportions, first relative to diminishing populations according to progression along the PIP, and then relative to the total population of caregivers (Figure 1).

○ P1 = Proportion of caregivers who ever heard of *Desta*, among all caregivers
○ P2 = Proportion of caregivers who ever received *Desta*, among only those caregivers who heard of *Desta*
○ P3 = Proportion of caregivers who ever fed *Desta*, among only those caregivers who heard of *Desta* and received it
○ P4 = Proportion of caregivers who fed *Desta* at least once within the last 14 days, among only those caregivers who heard of *Desta*, received it and ever fed it
○ PT = Proportion of caregivers who fed *Desta* at least once within the last 14 days, among all caregivers

### 2.5 Independent variables

Prior to this study, we conducted an analysis of the literature to identify factors that affect MNP adherence (Tumilowicz et al., 2017), which provided a theoretical basis for the selection of individual factors (independent variables) that may have a role in either facilitating or limiting MNP intervention outcomes. These potential determinants related to socio‐demographic factors, caregiver and child characteristics, IYC feeding practices, exposure to the intervention and perception‐of‐use factors. Table S2 lists all variables assessed in the analysis.

A socio‐economic status (SES) score was derived using principal component analysis (PCA) as per (Chasekwa et al., 2018) from variables covering household characteristics, asset ownership, water and sanitation. All variables were recoded as binary indicator variables, and those with frequencies <5% or >95% or >5% missing were excluded. This cutoff was used to offer discriminating power by excluding particularly uncommon and very common characteristics. Of the 60 variables initially considered, 39 met the inclusion criteria. To compute a SES score we first carried out polychoric PCA (with orthogonal varimax rotation) on the set of 39 variables and identified the first principal component, which explained 21% of the total variation. We then computed the SES score as the sum of standardized factor loadings from this first principal component. The resulting score was then validated internally and externally by examining trends in the proportion of ownership or presence of some assets across five quantiles of the score. Several variables were examined, some used in the construction of the index (internal validity) and others which were excluded (external validity).

IYC feeding practices were assessed using questions adapted from the WHO/United Nations International Children's Emergency Fund document on “Indicators of Infant and Young Child Feeding” (World Health Organization (WHO), 2008). Caregivers reported all foods the child consumed in the previous day and the frequency of feeding, and data collectors coded responses based on standard food groups. In the questionnaire the responses were recorded in 11 food groups that were subsequently recoded during data analysis into the seven WHO/United Nations International Children's Emergency Fund food groups of (1) grains/roots/tubers, (2) legumes/nuts, (3) dairy, (4) flesh foods, (5) eggs, (6) vitamin‐A‐rich fruits/vegetables and (7) other fruits/vegetables. From these scores, a child dietary diversity score was calculated and minimum dietary diversity defined as ≥4 of seven food groups, minimum meal frequency as solid/semi‐solid foods ≥2 times per day for breastfed infants 6–8 months, ≥3 times per day for breastfed children 9–23 months and ≥4 times per day for non‐breastfed children 6–23 months.

Household hunger was assessed using the Household Hunger Score (HHS; Ballard, Coates, Swindale, & Deitcher, 2011). The HHS is an indicator of household hunger and focuses on the food quantity dimension of food access. HHS consists of three occurrence questions and three frequency‐of‐occurrence questions. The HHS occurrence questions ask whether a specific condition associated with the experience of food insecurity ever occurred during the previous 30 days. The HHS frequency‐of‐occurrence questions ask how often a reported condition occurred during the previous 4 weeks: rarely, sometimes or often. Households with HHS > 1 are categorized as experiencing moderate or severe hunger.

We used polychoric PCA with orthogonal varimax rotation to compute perceptions‐of‐use latent factors and reduced the dimensions of the 25 perception‐of‐use questions (Chasekwa et al., 2018). Caregivers were asked without prompting to list all the positive changes, negative side effects and challenges that they perceived related to feeding *Desta* to their child. All 25 perception‐of‐use variables (Table S2) were recoded as binary indicator variables, and those with frequencies <5% or >95% or >5% missing responses were excluded as they were either too rare or too common to offer discriminating power. This resulted in 14 variables being included in the analysis (Table S3). Based on a scree‐plot of eigenvalues and examination of the factor loadings, we identified three principal components, which were conceptually meaningful. The three‐factor solution explained 45.3% of the total variance, 18.7% for factor 1, 14.4% for factor 2 and 12.2% for factor 3. A principal component score for each caregiver was then calculated by adding the standardized loadings for all variables in the set. Items that had loadings >0.15 on the first principal component (factor 1), which we refer to as “perceived positive outcomes,” included the number of positive changes caregivers reported, perceived physical growth, perceived increased strength, perceived increased activity, perceived increased immunity and perceived increased appetite. Items that significantly loaded on the second principal component (factor 2), referred to as “perceived negative side effects,” included the number of negative side effects reported by the caregiver, perceived loose stool/diarrhoea and perceived nausea/vomiting. Items that loaded on the third principal component (factor 3), referred to as “perceived challenges to feeding *Desta*,” included the number of challenges to feed *Desta* reported by caregiver, child rejection of food with *Desta* and perceived black stool. For ease of interpretation and comparison across factors, factor scores were standardized so that each has a mean of zero and standard deviation of one.

### 2.6 Data entry and statistical analysis

Double data entry was conducted by dedicated data entry clerks using Epi Info™ 7.2.1.0 (Dean et al., 2011). Subsequently, thorough consistency checks of the merged databases were conducted. Statistical weights applied during all analyses accounted for the differences between estimated EA populations used in first‐stage sampling and the actual EA population counted by the survey teams. Additional statistical weights were used in analyses of the entire sample to correct for different sampling fractions in different strata. We did not use a finite population correction factor in the design because the sampling fraction of the population was much lower than 5%. As a result, the *N* are the same as those reported in the actual survey. All analyses were carried out using Stata 14 (StataCorp, 2015) and accounted for survey design and weights. For continuous and categorical variables, means and proportions were compared across strata using a Wald or Pearson chi‐square test, respectively.

The association between each of the four conditional outcomes (P1, P2, P3, and P4) and outcome among all caregivers (PT) with independent variables was estimated using generalized linear models with a Poisson log‐link function and cluster‐robust standard error estimates. The coefficients for each step (P1–P4) are the effects as one moves from P1 (ever heard) through P4 (fed *Desta* at least once within the last 14 days). Thus, changes in a coefficient over the P reflect changes in its association as one moves along the PIP. A final model for PT was estimated, which gave the adjusted relative risk of continued feeding among all caregivers. To construct multivariable models, first bivariate analyses were explored with each independent predictor variable (Table S4). A full multivariable model was then constructed using all predictors with *P* < 0.25 in bivariate analyses or with a strong theoretical basis for inclusion. In sensitivity analyses, parsimonious models were identified using forward step‐wise selection permitting all covariates to enter at *P* < 0.25. The adequacy of the parsimonious models was assessed against the full models using Akaike information criterion and Bayesian information criterion. The full models were found to adequately explain the data for each outcome.

### 2.7 Ethical considerations

The survey protocol was approved by the Ethiopia National Research Ethics Committee, clearance number IEHD/250/12/08. Written informed consent was sought from the caregiver of each selected child. Children with a mid‐upper arm circumference <110 mm were referred to the nearest health facility following MOH protocol for the treatment of severe acute malnutrition.

## 3 RESULTS

Descriptive data of the survey sample show differences among strata, with the largest contrasts being between the urban stratum and the two rural strata of Amhara and Tigray (Table 1). Although most caregivers from the urban stratum were classified in the highest SES tercile, most caregivers from rural strata were distributed between the middle and lower SES terciles. Compared with caregivers from the rural strata, caregivers in the urban stratum were more educated, and a lower proportion perceived the distance to access HEW and health services as “very long.” Children in the urban stratum had higher dietary diversity and lower breastfeeding prevalence. In the urban stratum, a lower proportion of caregivers reported learning about *Desta* from HEW or health centre staff and receiving interpersonal counselling on *Desta*. The perceptions of both positive outcomes and negative side effects were highest in the Amhara rural stratum compared with the other strata, and the perceived challenges to feed *Desta* was highest in the urban stratum.

**Table 1:** Characteristics of survey population, pilot design features and perception‐of‐use variables

	Total (114 clusters)		Amhara rural (40 clusters)		Tigray rural (40 clusters)		Urban (34 clusters)		*P*‐value
Variable	*N*	Mean/percentage (95% CI)	*N*	Mean/percentage (95% CI)	*N*	Mean/percentage (95% CI)	*N*	Mean/percentage (95% CI)	Among strata
*Socio‐demographic*									
SES score^a^, mean	1,916	0 (−0.23, 0.23)	655	−0.63 (−0.90, −0.36)	683	−0.30 (−0.72, 0.12)	578	5.46 (4.82, 6.10)	<0.001
SES tercile:									<0.001
Upper, %	633	15.3 (11.5, 20.2)	39	6.0 (2.3, 14.8)	75	12.4 (6.3, 22.8)	519	89.6 (83.1, 93.8)
Middle, %	642	41.1 (37.2, 45.0)	291	44.8 (38.8, 50.9)	303	43.0 (37.8, 48.4)	48	8.2 (5.0, 13.1)
Lower, %	641	43.6 (39.2, 48.0)	325	49.2 (43.1, 55.3)	305	44.7 (37.1, 52.5)	11	2.2 (0.1, 5.3)
Little to no hunger in HH, %	1,895	98.9 (98.1, 99.3)	645	98.5 (96.9, 99.2)	679	99.5 (98.6, 99.8)	571	98.8 (97.3, 99.5)	0.113
Caregivers who perceived distance to access a HEW was very long, %	201	13.1 (9.5, 17.8)	85	12.8 (7.7, 20.7)	115	16.4 (11.2, 23.3)	1	0.2 (0, 1.2)	0.042
Caregivers who perceived distance to health centre was very long, %	347	22.7 (17.9, 28.4)	175	25.3 (18.0, 34.4)	169	23.9 (17.1, 32.3)	3	0.5 (0.2, 1.5)	0.010
*Caregiver*									
Age of caregiver (y), mean	1,915	29.1 (28.6, 29.6)	654	28.6 (27.9, 29.3)	683	29.9 (29.1, 30.7)	578	29.1 (28.4, 29.8)	0.057
Caregiver <5‐y education, %	1,120	65.8 (61.3, 69.9)	491	74.5 (68.4, 79.8)	402	58.7 (50.7, 66.3)	227	40.3 (34.5, 46.3)	<0.001
Caregiver highest level of education:									
None/illiterate, %	809	48.0 (43.4, 52.6)	350	53.9 (47.2, 60.5)	299	43.9 (36.6, 51.5)	160	27.8 (22.4, 33.8)	<0.001
Informal education, literate, %	86	5.1 (3.7, 7.0)	50	7.4 (5.0, 10.9)	14	1.9 (1.0, 3.9)	22	3.9 (2.2, 6.9)	
Formal education, %	1,021	46.9 (42.5, 51.4)	255	38.6 (32.8, 44.8)	370	54.2 (46.3, 61.8)	396	68.4 (62.4, 73.8)	
*Child*									
Age of child (mo), mean	1,916	14.4 (14.1, 14.7)	655	14.2 (13.7, 14.6	683	14.6 (14.2, 15.1)	578	14.5 (14.1, 14.9)	0.305
Child sex female, %	965	50.4 (47.6, 53.1)	314	47.7 (43.9, 51.6)	372	54.6 (50.0, 59.2)	279	48.7 (44.9, 52.6)	0.027
*Infant and young child feeding*									
Currently breastfed, %	1,666	88.7 (86.5, 90.6)	583	88.9 (85.0, 91.8)	615	90.2 (88.0, 92.0)	468	81.3 (76.3, 85.5)	0.025
Child dietary diversity score (CHDDS)^b^, mean	1,916	2.04 (1.97, 2.11)	655	2.05 (1.96, 2.15)	683	1.94 (1.81, 2.07)	578	2.39 (2.22, 2.57)	<0.001
Child minimum dietary diversity score (CDDS≥4)^b^, %	167	6.2 (4.9, 7.7)	33	4.9 (3.2, 7.3)	37	5.5 (3.7, 8.1)	97	17.6 (13.6, 22.5)	<0.001
Child ate solid or semi‐solid food in 24 h preceding survey, %	1,824	95.1 (93.6, 96.3)	629	95.6 (93.0, 97.3)	645	94.5 (92.4, 96.0)	550	95.3 (92.9, 96.9)	0.535
Child meal frequency, mean	1,841	2.97 (2.85, 3.09)	630	2.94 (2.75, 3.12)	653	2.98 (2.81, 3.14)	558	3.19 (2.99, 3.39)	0.160
Child minimum meal frequency^b^, %	1,265	68.3 (64.2, 72.1)	439	68.3 (61.7, 74.3)	447	68.1 (62.9, 72.9)	379	68.7 (62.4, 74.4)	0.983
*Pilot design features*									
Number of times caregiver met HEW or health centre staff in 3‐mo preceding survey, mean	1,916	1.90 (1.75, 2.05)	655	1.77 (1.54, 2.01)	683	2.10 (1.89, 2.32)	578	1.77 (1.46, 2.08)	0.071
Caregivers who received neither feeding nor Desta counselling, %	311	12.7 (10.9, 14.8)	89	13.3 (10.8, 16.3)	57	8.6 (6.0, 12.3)	165	27.2 (20.1, 35.7)	<0.001
Caregivers who received feeding counselling only, %	417	21.4 (18.4, 24.7)	162	23.5 (18.8, 29.0)	126	18.2 (14.6, 22.6)	129	21.8 (17.3, 27.2)	
Caregivers who received Desta counselling only, %	50	2.4 (1.6, 3.7)	12	2.1 (1.0, 4.4)	17	2.5 (1.4, 4.4)	21	3.9 (2.3, 6.5)	
Caregivers who received both feeding and Desta counselling, %	1,138	63.5 (59.6, 67.2)	392	61.0 (54.8, 66.9)	483	70.6 (65.7, 75.2)	263	47.1 (37.5, 56.9)	
*Perceptions of use and outcomes scores* c									
Perceived positive outcomes score, mean	1,093	0 (−0.16, 0.16)	370	.02 (−0.17, 0.21)	456	0 (−0.29, 0.29)	267	−0.18 (−0.56, 0.20)	0.638
Perceived negative side effects score, mean	1,093	0 (−0.14, 0.14)	370	0.17 (−0.08, 0.41)	456	−0.23 (−0.39, −0.06)	267	0.12 (−0.09, 0.33)	0.009
Perceived challenges to feed Desta score, mean	1,093	0 (−0.12, 0.12)	370	0.10 (−0.10 0.29)	456	−0.15 (−0.31, 0)	267	0.20 (−0.01, 0.39)	0.013

*Note*. HEW: health extension worker.

a Socio‐economic status (SES) score computed using principal component analysis following Demographic and Health Survey methodology (Chasekwa et al., 2018).

b World Health Organization (WHO; 2008).

c Perceptions of use and outcomes scores derived from factor analysis.

**Figure 2:**
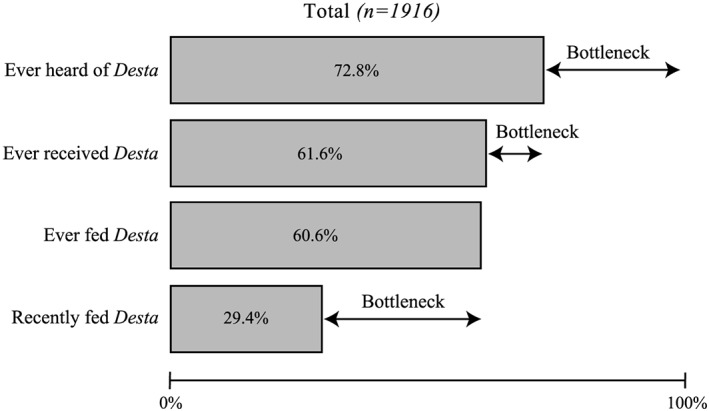
Bottlenecks in coverage and adherence outcomes among all caregivers (*N* = 1916) reflective of transfer of information and *Desta* from the program to the caregiver and household delivery system. *Desta*, brand of micronutrient powder used in the trial. “Ever heard of *Desta*,” proportion of all caregivers with children 6–23 months of age who ever heard of *Desta*. “Ever received *Desta*,” proportion all caregivers with children 6–23 months of age who ever received *Desta.* “Ever fed *Desta*,” proportion of all caregivers with children 6–23 months of age who ever fed *Desta* to the child. “Recently fed *Desta*,” proportion of all caregivers with children 6–23 months of age who fed *Desta* to the child at least one time in the 14 days preceding the survey

Analysis of bottlenecks using the Tanahashi model of coverage and adherence outcomes among all caregivers (*N* = 1916) showed that the largest bottlenecks occurred at the step of hearing about *Desta* and in the step from initiating feeding *Desta* to having recently fed it (Figure 2). The proportions of caregivers achieving each step in the PIP significantly varied across strata (Table 2). The largest magnitude of differences across strata occurred at the step of hearing about *Desta*: 43.3% of caregivers in the urban strata, 30.0% in Amhara and 19.6% in Tigray did not learn about *Desta*. Notably, nearly all caregivers who received *Desta* initiated feeding it to their child.

**Table 2:** *Desta* coverage and adherence outcomes among all caregivers (*N* = 1916)

	Total population	Bottleneck →	Ever heard of *Desta*	Bottleneck →	Ever received *Desta*	Bottleneck →	Ever fed *Desta*	Bottleneck →	Fed *Desta* within the last 14 days
Total and strata	%	%	% (95% CI)	%	% (95% CI)	%	% (95% CI)	%	% (95% CI)
Total (*N* = 1916)	100.0	27.2	72.8 (68.7, 76.5)	11.2	61.6 (57.5, 65.5)	1.0	60.6 (56.6, 64.4)	31.2	29.4 (25.8, 33.3)
Amhara rural (*N* = 655)	100.0	30.0	70.0 (63.3, 75.9)	10.6	59.4 (53.0, 65.5)	1.3	58.1 (51.9, 64.0)	33.1	25.0 (19.7, 31.1)
Tigray rural (*N* = 683)	100.0	19.6	80.4 (75.4, 84.5)	12.8	67.6 (61.9, 72.8)	0.5	67.1 (61.5, 72.3)	29.7	37.4 (32.3, 42.9)
Urban (*N* = 578)	100.0	43.3	56.7 (47.0, 65.9)	7.9	48.8 (39.7, 58.0)	1.2	47.6 (38.4, 57.0)	25.6	22.0 (16.3, 29.0)
*P*‐value among strata	‐	‐	<0.001	‐	0.010	‐	0.005	‐	0.001

The most frequently cited reasons for not having recently fed *Desta* werethe caregiver finished the supply and did not obtain additional supply (36.1%), perceived child rejection of food mixed with *Desta* (22.9%) and perceived negative side effects as a result of feeding *Desta* (9.1%; Table 3). More caregivers from Amhara rural (44.1%, 95% CI: 34.3, 54.4) than Tigray rural (27.1%, 95% CI: 19.5, 36.3) and urban (15.6%, 95% CI: 9.9, 23.9) strata reported not obtaining additional supply of *Desta* (*P* = 0.002). As children aged, the proportion of caregivers who reported not obtaining an additional supply increased, although the differences among the age groups was not statistically significant (*P* = 0.258): 29.1% (95% CI: 14.7, 49.5) of caregivers of infants 6–11 months, 33.4% (95% CI: 26.4, 41.2) of caregivers of children 12–17 months and 41.6% (95% CI: 32.5, 51.3) of caregivers of children 18–23 months. Overall, 22.9% (95% CI: 18.5, 27.9) of caregivers who had stopped feeding *Desta* did so because they perceived the child rejected food mixed with *Desta*, with no significant differences in proportions of caregivers reporting this problem across strata or age group.

**Table 3:** Reasons for not feeding Desta among caregivers who did not feed Desta to child in 14 days previous the survey (*N* = 556)

	Total		Amhara rural		Tigray rural		Urban		*P*‐value	6–11 mo		12–17 mo		18–23 mo		*P*‐value
Variable	*N*	Percentage (95% CI)	*N*	Percentage (95% CI)	N	Percentage (95% CI)	*N*	Percentage (95% CI)	Among strata	*N*	Percentage (95% CI)	*N*	Percentage (95% CI)	*N*	Percentage (95% CI)	Among age groups
Caregiver finished supply and did not obtain additional supply	173	36.1 (29.7, 43.0)	94	44.1 (34.3, 54.4)	56	27.1 (19.5, 36.3)	23	15.6 (9.9, 23.9)	0.002	19	29.1 (14.7, 49.5)	77	33.4 (26.4, 41.2)	77	41.6 (32.5, 51.3)	0.258
Child rejected food mixed with Desta	140	22.9 (18.5, 27.9)	46	21.4 (15.4, 28.9)	47	23.5 (17.1, 31.3)	47	32.7 (25.5, 40.9)	0.296	25	25.5 (15.1, 39.7)	67	25.5 (19.7, 32.2)	48	19.0 (13.6, 26.0)	0.303
Perceived negative side effects	55	9.1 (6.4, 12.7)	19	8.9 (5.3, 14.4)	15	8.3 (4.6, 14.6)	21	15.2 (9.3, 23.8)	0.412	8	9.4 (3.8, 21.1)	31	12.3 (7.3, 19.9)	16	5.4 (3.0, 9.3)	0.303

*Note*. CI: confidence interval.

Examining the changes of a single independent variable across levels of coverage and adherence outcomes from P1 to P4 gives insights about their associations with bottlenecks as one moves along the PIP (Table 4). In other words, one can identify variables that are associated with increased or decreased outcomes as one moves across the PIP. Sometimes, the direction of variables' association may go in opposite directions at different steps and thus can cancel each other out in PT. Below we discuss independent variables with significance *P* < 0.05 as one moves along the PIP from P1 to P4, as well as their overall effects reflected in PT (Table 4):

**Table 4:** Determinants of coverage and adherence outcomes across program impact pathway steps

	P1^a^		P2^b^		P3^c^		P4^d^		PT^e^	
Variable	Multivariable Adj RR (*N* = 1915)		Multivariable Adj RR (*N* = 1315)		Multivariable Adj RR (*N* = 1110)		Multivariable Adj RR (*N* = 1092)		Multivariable Adj RR (*N* = 1915)	
Factor	RR (95% CI)	*P*‐value	RR (95% CI)	*P*‐value	RR (95% CI)	*P*‐value	RR (95% CI)	*P*‐value	RR (95% CI)	*P*‐value
Stratum (Amhara rural)	1.00 (ref)	‐	1.00 (ref)	‐	1.00 (ref)	‐	1.00 (ref)	‐	1.00 (ref)	‐
Tigray rural	1.03 (0.98, 1.09)	0.261	0.96 (0.92, 1.01)	0.152	1.01 (1.00, 1.03)	0.099	1.31 (1.08, 1.57)	0.005	1.28 (1.04, 1.58)	0.021
Urban	0.89 (0.78, 1.02)	0.093	0.98 (0.89, 1.08)	0.655	1.00 (0.97, 1.02)	0.799	1.15 (0.87, 1.53)	0.317	1.11 (0.82, 1.51)	0.487
*Socio‐demographic*										
SES quantile (lowest)	1.00 (ref)	‐	1.00 (ref)	‐	1.00	‐	1.00 (ref)	‐	1.00 (ref)	‐
Middle	1.02 (0.98, 1.05)	0.404	1.03 (0.98, 1.07)	0.235	1.02 (1.00, 1.04)	0.097	0.96 (0.84, 1.11)	0.606	1.01 (0.87, 1.18)	0.896
Upper	1.07 (0.96, 1.19)	0.217	1.07 (0.97, 1.17)	0.172	1.01 (0.99, 1.03)	0.557	0.92 (0.75, 1.14)	0.452	0.98 (0.77, 1.24)	0.844
Household hunger (HHS > 1, indicating moderate or severe hunger)^f^	0.95 (0.89, 1.02)	0.139	1.10 (1.03, 1.19)	0.009	1.02 (1.01, 1.04)	0.009	1.11 (0.60, 2.03)	0.742	1.21 (0.65, 2.26)	0.551
*Caregiver*										
Caregiver's age (y)	1.00 (1.00, 1.00)	0.331	1.00 (1.00, 1.00)	0.535	1.00 (1.00, 1.00)	0.561	0.99 (0.98, 1.00)	0.100	0.99 (0.98, 1.00)	0.088
Caregiver education (<5 y)^g^	0.99 (0.95, 1.04)	0.786	0.96 (0.92, 0.99)	0.026	1.00 (0.98, 1.02)	0.945	1.06 (0.89, 1.26)	0.542	1.01 (0.84, 1.21)	0.949
*Child*										
Age of child (6–11 mo)	1.00 (ref)	‐	1.00 (ref)	‐	1.00 (ref)	‐	1.00 (ref)	‐	1.00 (ref)	‐
12–17 mo	0.96 (0.91, 1.01)	0.103	1.21 (1.14, 1.28)	<0.001	1.02 (0.99, 1.06)	0.132	0.68 (0.57, 0.82)	<0.001	0.82 (0.68, 0.98)	0.033
18–23 mo	0.94 (0.88, 0.99)	0.019	1.19 (1.12, 1.27)	<0.001	1.01 (0.98, 1.05)	0.488	0.62 (0.51, 0.77)	<0.001	0.73 (0.58, 0.92)	0.007
Child sex (female)^h^	1.02 (0.98, 1.06)	0.315	1.00 (0.96, 1.04)	0.918	1.00 (0.98, 1.02)	0.931	1.14 (1.01, 1.29)	0.037	1.13 (1.00, 1.29)	0.055
*Infant and young child feeding*										
Currently breastfed (yes)^i^	1.00 (0.93, 1.07)	0.933	0.98 (0.93, 1.03)	0.382	0.99 (0.97, 1.00)	0.034	0.91 (0.73, 1.13)	0.377	0.87 (0.69, 1.09)	0.227
Child ate solid or semi‐solid food in 24‐h preceding survey^j^	1.06 (0.93, 1.22)	0.382	1.70 (1.19, 2.43)	0.004	1.15 (0.94, 1.41)	0.179	1.14 (0.69, 1.91)	0.602	2.49 (1.31, 4.72)	0.006
Child dietary diversity score (CDDS)	1.01 (0.99, 1.03)	0.395	0.98 (0.96, 1.00)	0.019	1.00 (1.00, 1.01)	0.146	0.97 (0.90, 1.05)	0.456	0.96 (0.88, 1.04)	0.269
*Pilot design features*										
Number of times caregiver met with HEW or health centre staff in 3‐mo preceding survey	1.00 (0.99, 1.01)	0.481	1.00 (0.99, 1.02)	0.415	1.00 (0.99, 1.00)	0.242	1.13 (1.10, 1.17)	<0.001	1.13 (1.10, 1.17)	<0.001
Caregiver perception of distance to access a HEW is very long (yes)^k^	0.96 (0.88, 1.04)	0.293	0.99 (0.92, 1.07)	0.832	1.03 (1.00, 1.07)	0.058	1.11 (0.85, 1.45)	0.444	1.13 (0.87, 1.47)	0.366
Caregiver received neither feeding nor Desta counselling	1.00 (ref)	‐	1.00 (ref)	‐	1.00 (ref)	‐	1.00 (ref)	‐	1.00 (ref)	‐
Caregiver received feeding counselling only	1.06 (0.70, 1.62)	0.781	0.62 (0.21, 1.81)	0.383	1.00 (0.98, 1.02)	0.883	1.38 (0.21, 9.19)	0.735	0.94 (0.08, 11.16)	0.960
Caregiver received Desta counselling only	5.06 (3.47, 7.38)	<0.001	4.89 (2.13, 11.24)	<0.001	0.98 (0.94, 1.02)	0.333	0.34 (0.05, 2.45)	0.280	10.79 (1.22, 95.95)	0.032
Caregiver received both feeding and Desta counselling	5.08 (3.48, 7.41)	<0.001	6.59 (2.97, 14.61)	<0.001	0.99 (0.96, 1.01)	0.250	1.23 (0.21, 7.38)	0.817	54.14 (7.32, 400.59)	<0.001

*Note*. CI: confidence interval; HHS: Household Hunger Score; RR: relative risk; SES: socio‐economic status.

a P1 = Proportion of caregivers who ever heard of Desta, among all caregivers.

b P2 = Proportion of caregivers who ever received Desta, among only those caregivers who heard of Desta.

c P3 = Proportion of caregivers who ever fed Desta, among only those caregivers who heard of Desta and received it.

d P4 = Proportion of caregivers who fed Desta at least once within the last 14 days, among only those caregivers who heard of Desta, received it and ever fed it.

e PT = Proportion of caregivers who fed Desta at least once within the last 14 days, among all caregivers.

f Referent is HHS is ≤1, indicating no or little hunger.

g Referent is caregiver education ≥5 years.

h Referent is child sex is male.

i Referent is child not currently breastfed.

j Referent is child did not eat solid or semi‐solid food in 24‐hr preceding survey.

k Referent is caregiver perception of distance to access a health extension workers (HEWs) is short or long but manageable.

### 3.1 Stratum

Among caregivers who ever fed *Desta*, caregivers from Tigray rural stratum were about 30% more likely to have recently fed it to their child than those from Amhara rural stratum (P4, ARR (95% CI): 1.31 (1.08, 1.57) *P* = 0.005), and this association remained evident in PT (ARR (95% CI): 1.28 (1.04, 1.58) *P* = 0.021).

### 3.2 Household hunger (HHS > 1, indicating moderate or severe hunger)

Caregivers from households reporting moderate or severe hunger were more likely to have received *Desta* (P2, ARR [95% CI]: 1.10 [1.03, 1.19] *P* = 0.009) and initiated feeding it to their children (P3, ARR (95% CI): 1.02 (1.01, 1.04) *P* = 0.009). Household hunger was not found to be associated with any other step in the PIP or PT.

### 3.3 Caregiver education

Among caregivers who heard of *Desta*, those with <5 years of education were less likely to have received *Desta* than caregivers with ≥5 years of education (P2, ARR (95% CI): 0.96 (0.92, 0.99) *P* = 0.026). Caregiver education was not found to be associated with any other step in the PIP or PT.

### 3.4 Child age

Caregivers of children 18–23 months were less likely to have heard about *Desta* than caregivers of children 6–11 months (P1, ARR [95% CI]: 0.94 [0.88, 0.99] *P* = 0.019). Among caregivers who heard of *Desta*, those with children 12–17 months and 18–23 months were about 20% (P2, ARR [95% CI]: 1.21 [1.14, 1.28] *P* < 0.001 and ARR [95% CI]: 1.19 [1.12, 1.27] *P* < 0.001, respectively) more likely to have received it than caregivers of children 6–11 months. Among children whose caregivers initiated feeding *Desta*, children 12–17 months were 32% (P4, ARR [95% CI]: 0.68 [0.57, 0.82] *P* < 0.001) and children 18–23 months 38% (P4, ARR [95% CI]: 0.62 [0.51, 0.77] *P* < 0.001) less likely to have been recently fed *Desta* than children 6–11 months. When considering the cumulative effect of child age across all steps, children 12–17 months were 18% (PT, ARR [95% CI]: 0.82 [0.68, 0.98] *P* = 0.033) and children 18–23 months 27% (PT, ARR [95% CI]: 0.73 [0.58, 0.92] *P* = 0.007) less likely to have been recently fed *Desta* than children 6–11 months.

### 3.5 Child sex

Among children whose caregivers initiated feeding *Desta*, girls were 14% more likely to have been recently fed *Desta* than boys (P4, ARR [95% CI]: 1.14 [1.01, 1.29] *P* = 0.037). In the cumulative effect of sex across all steps, girls were 13% more likely to have been recently fed *Desta* than boys (PT, ARR [95% CI]: 1.13 [1.00, 1.29] *P* = 0.055).

### 3.6 Breastfeeding

Caregivers who received *Desta* and initiated feeding it were slightly less likely to be currently breastfeeding (P3, ARR [95% CI]: 0.99 [0.97, 1.00] *P* = 0.034). Breastfeeding was not found to be associated with any other step in the PIP or PT.

### 3.7 Child ate solid or semi‐solid food in 24‐hr preceding survey

Expectedly, among caregivers who heard about *Desta*, those with children who consumed solid or semi‐solid foods in the 24‐hr preceding the survey were 70% more likely to have received it (P2, ARR [95% CI]: 1.70 [1.19, 2.43] *P* = 0.004). When considering the cumulative effect across steps, children who had consumed solid or semi‐solid foods were considerably more likely to have been recently fed *Desta* (PT, ARR [95% CI]: 2.49 [1.31, 4.72] *P* = 0.006).

### 3.8 Child dietary diversity

Increasing child dietary diversity was slightly negatively associated with having received *Desta* among those who heard about it (P2, ARR [95% CI]: 0.98 [0.96, 1.00] *P* = 0.019). Child dietary diversity was not found to be associated with any other step in the PIP or PT.

### 3.9 Frequency of contact with HEW or health centre staff

Among caregivers who initiated feeding *Desta*, for every additional time that a caregiver met with the HEW or health centre staff in the 3 months prior to the survey, they were 13% more likely to have recently fed it (P4, ARR [95% CI]: 1.13 [1.10, 1.17] *P* < 0.001). The effect remained the same when considering the cumulative effect of contacts with HEW or health centre staff across all steps (P4, ARR [95% CI]: 1.13 [1.10, 1.17] *P* < 0.001).

### 3.10 Counselling from HEW or health centre staff

Receiving counselling from a HEW or health centre staff that included *Desta* as a topic was strongly positively associated with hearing about it (P1, ARR [95% CI]: 5.06 [3.47, 7.38] *P* < 0.001 and ARR [95% CI]: 5.06 [3.48, 7.41] *P* < 0.001). Among caregivers who heard of *Desta*, receiving counselling from a HEW or health centre staff that included *Desta* was strongly positively associated with receiving it (P2, ARR [95% CI]: 4.89 [2.13, 11.24] *P* < 0.001 and ARR [95% CI]: 6.59 [2.97, 14.61] *P* < 0.001). When considering the cumulative effect of counselling across all steps, caregivers who received counselling from a HEW or health centre staff that included *Desta* were substantially more likely to have recently fed it (PT, ARR [95% CI]: 10.79 [1.22, 95.95] *P* = 0.032 and ARR [95% CI]: 54.14 [7.32, 400.59] *P* < 0.001).

### 3.11 Perception of use and outcomes scores

Among caregivers who initiated feeding *Desta*, one Z‐score increase in the caregivers' perception that *Desta* produced positive changes in children was associated with a 14% increase in the likelihood of having recently fed it (ARR [95% CI]: 1.14 [1.10, 1.19] *P* < 0.001). The addition of perceptions of use and outcomes scores derived from factor analysis to the multivariable model did not substantially change the other determinants' effects (Table 5).

**Table 5:** Determinants including perceptions of use and outcome scores of P4^a^

	P4	
Variable	Multivariable Adj RR (*N* = 1092)	
Factor	RR (95% CI)	*P*‐value
Stratum (Amhara rural)	1.00 (ref)	
Tigray rural	1.27 (1.06, 1.53)	0.011
Urban	1.18 (0.91, 1.53)	0.204
*Socio‐demographic*		
SES quantile (lowest)	1.00 (ref)	
Middle	0.97 (0.85, 1.10)	0.616
Upper	0.93 (0.76, 1.13)	0.456
Household hunger (HHS > 1, indicating moderate or severe hunger)^b^	0.99 (0.98, 1.00)	0.085
*Caregiver*		
Caregiver's age (y)	0.99 (0.98, 1.00)	0.085
Caregiver education (<5 y)^c^	1.04 (0.87, 1.23)	0.681
*Child*		
Age of child (6–11 mo)	1.00 (ref)	
12–17 mo	0.66 (0.56, 0.79)	<0.001
18–23 mo	0.60 (0.49, 0.74)	<0.001
Child sex (female)^d^	1.17 (1.03, 1.32)	0.014
*Infant an young child feeding*		
Currently breastfed (yes)^e^	0.96 (0.78, 1.18)	0.702
Child ate solid or semi‐solid food in 24‐hr preceding survey^f^	1.15 (0.70, 1.87)	0.583
Child dietary diversity score (CDDS)	0.97 (0.90, 1.05)	0.476
*Pilot design features*		
Number of times caregiver met with HEW or health centre staff in 3‐mo preceding survey	1.11 (1.07, 1.14)	<0.001
Caregiver perception of distance to access a HEW is very long (yes)^g^	1.01 (0.77, 1.33)	0.917
Caregiver received neither feeding nor Desta counselling	1.00 (ref)	
Caregiver received feeding counselling only	1.30 (0.19, 8.80)	0.788
Caregiver received Desta counselling only	0.36 (0.06, 2.32)	0.280
Caregiver received both feeding and Desta counselling	1.16 (0.22, 6.18)	0.863
*Perceptions of use and outcomes scores* h		
Perceived positive outcomes score (factor 1)	1.14 (1.10, 1.19)	<0.001
Perceived negative side effects score (factor 2)	0.98 (0.93, 1.03)	0.465
Perceived challenges to feed *Desta* score (factor 3)	0.95 (0.89, 1.01)	0.091

*Note*. CI: confidence interval; HHS: Household Hunger Score; RR: relative risk; SES: socio‐economic status.

a Proportion of caregivers who fed *Desta* at least once within the last 14 days, among only those caregivers who heard of *Desta*, received it and ever fed it (P4).

b Referent is HHS is ≤1, indicating no or little hunger.

c Referent is caregiver education ≥5 years.

d Referent is child sex is male.

e Referent is child not currently breastfed.

f Referent is child did not eat solid or semi‐solid food in 24‐hr preceding survey.

g Referent is caregiver perception of distance to access a HEW is short or long but manageable.

h Perceptions of use and outcomes scores derived from factor analysis.

## 4 DISCUSSION

The process of adherence to health interventions has been articulated in a model by Vrijens et al. (Vrijens et al., 2012). In this model, the process starts with “prescription” of the intervention, which occurs when individuals learn about the intervention, receive it and agree to try it. The process continues with “initiation,” consisting of trying the intervention and then continuing to practice the new behaviour. The final phase is “continuation” or “persistence,” defined as the period between initiation and the last dose. The study presented here was designed to permit us to differentiate the steps through which messages, and *Desta* were transferred from program implementers to caregivers and from caregivers to their children, so that we could examine the determinants of each step in the process of adherence.

Using this approach, we discovered the central role and unexpected pattern that child age plays in affecting MNP coverage and adherence outcomes. Despite caregivers of children >11 months being more likely to have received *Desta*, after 1 year of age the probability of caregivers continuing to feed it plummeted. Older children had a longer exposure period to the program delivery system, so it could be expected that their caregivers were more likely to have ever received *Desta*. However, as one moves to the phase of continuation or persistence, we found that after infancy, children were progressively less likely to have been fed *Desta* recently. Possible explanations for this finding include:

1. Over time developmental changes and illness may affect children's acceptance of food mixed with Desta. The survey results showed that 22.9% of caregivers who stopped feeding *Desta* did so because they perceived that their children refused food mixed with it (Table 3). Our findings from the FES, which was conducted in a subsample of caregivers who participated in the trial (Pelto et al., 2019), provide insights for the interpretation of this result. In the FES sample, nine caregivers spontaneously said that rejection of food mixed with *Desta* was the reason for stopping it, but all except one reported that initially, and even for periods of up to 9 months before they began to refuse it, the child had accepted it. These findings may be attributable to a natural evolution of taste preferences. Many children in the second and third years of life enter a “neophobic” phase during which some previously liked foods are no longer accepted, and the introduction of new foods becomes difficult (Nicklaus, 2009). With respect to MNP, many nutrition professionals have adopted the view that if the food is prepared following recommendations, infants and young children cannot differentiate food that contains MNP. However, a recently published sensory evaluation study of MNP suggests there may be exceptions to this assumption (Sutrisna, Vossenaar, Izwardy, & Tumilowicz, 2017). It is plausible that some children, particularly after 12 months of age, may be negatively reacting to organoleptic changes of food mixed with MNP. At the same time, several caregivers interviewed in the FES cited general poor appetite and diarrhoea in addition to refusing food with *Desta*. Although caregivers may have perceived that the child rejected food because it contained MNP, there could be other explanations such as illness.
2. Problems with obtaining a new supply. It is possible that caregivers of older children were more likely to have stopped using *Desta* because they were less likely to obtain a new supply (Table 3). According to the MOH vaccination schedule, the last vaccine (measles) for children under 5 years of age is administered at 9 months of age, and caregivers often do not seek other routine services, such as growth monitoring and promotion after immunizations are completed (Bilal, Moser, Blanco, Spigt, & Dinant, 2014; Federal Ministry of Health, 2015). Moreover, HEW prioritize home visits for children who need immunizations or medical treatment. Unless a special trip was undertaken, older children whose caregivers were no longer seeking routine services would not have received a new supply of *Desta*. Caregivers were provided 30 sachets of *Desta* and instructed to feed it every other day to their child. Caregivers of infants 6–11 months were likely to still have their original supply of *Desta*. This inference is supported by themes that emerged from the FES narratives related to caregivers' difficulties to obtain *Desta* refills (Pelto et al., 2019).

Frequent contacts with HEW or health staff (i.e. frontline workers) fostered continued use of MNP. FES results found that inter‐personal communication by the HEW and cooking demonstrations played a central role in mothers' reports pertaining to knowledge, confidence and problem‐solving strategies to prepare and feed food with *Desta* (Pelto et al., 2019). In the FES sample, six of the nine caregivers who reported problems with child refusal of food with Desta sought advice from HEW.

Caregivers' positive perceptions concerning the use of *Desta* also facilitated continued use. Our results are consistent with previous studies, which have found correlations between perceived positive changes in children and increased MNP coverage and adherence, and no correlations between perceived negative side effects and discontinuation of MNP (Jefferds et al., 2015; Mirkovic et al., 2016; Tumilowicz et al., 2017). The influence of negative side effects on adherence appears to depend on the counselling received by caregivers (Loechl et al., 2009; Tripp et al., 2011). When caregivers are forewarned about the possible negative side effects of MNP (i.e. changes in stool including darker than usual, mild diarrhoea or a mild form of constipation), experiencing them does not appear to deter continued MNP use, and, paradoxically, their appearance may even encourage continued use (Loechl et al., 2009; Mirkovic et al., 2016; Tripp et al., 2011). Caregivers in Ethiopia were advised that in the first few days of taking *Desta*, they may observe changes in the child's stool, which will usually disappear after a period of 4–5 days (Table SS1).

Shifting attention to the effect of sex, the positive association of being a female child on recent feeding of *Desta* is not explainable based on available evidence. The prevalence of stunting among Ethiopian girls 0–23 months of age has been reported to be lower than that of boys, but the difference disappears after the second year of life (Woodruff et al., 2017). Sex and gender differences in IYC feeding practices and growth are evident across very different national and cultural contexts, including Senegal (Bork & Diallo, 2017), Guatemala (Tumilowicz, Habicht, Pelto, & Pelletier, 2015) and the Philippines (Adair & Guilkey, 1997; Popkin et al., 1990). Indigenous mothers in Guatemala were found to perceive boys as hungrier and requiring earlier and more frequent complementary feeding compared with girls. In contrast to the findings of the current study, we expect Guatemalan indigenous boys would be fed food with MNP more often than girls. We encourage future studies in Ethiopia and other contexts to explore the interactions of biological‐cultural‐ecological factors that produce differences between sexes in IYC feeding and growth patterns. A model for this type of research has been previously proposed (Tumilowicz et al., 2015).

The main limitation of this study is its cross‐sectional design. A stronger case for attributing the relative longitudinal influence of specific factors to caregivers' interaction with the program delivery system and adherence to MNP recommendations requires a longitudinal cohort study design. Following children over a period of months would allow for deeper exploration and testing of hypotheses regarding why the use of MNP declines with age. It is also required to construct a more accurate indicator of effectiveness coverage or adherence to recommendations. Despite the design limitation, examining the steps in the PIP helps to interpret the results of the combined model (PT) and pinpoint at which steps in the PIP opportunities lie for program implementers to improve coverage and adherence outcomes. The analytic methodology presented in this study, as well as the emergent, substantive findings of the factors associated with key bottlenecks, can be used to inform the design of epidemiological studies and evaluations of MNP programs.

Our findings indicate that caregivers face challenges to continue feeding MNP through their children's second year of life as they experience periods of illness and poor appetite, progress through developmental stages that affect feeding behaviours and stop routine attendance at health services. Notwithstanding these challenges, frequent contacts with frontline workers fostered continued use of MNP, likely through ensuring caregivers have supply of MNP, alerting caretakers to positive changes and potential negative side effects in the child, and helping address MNP and IYC feeding difficulties. This study contributes to growing evidence of the critical role of frontline workers in MNP adherence (Reerink et al., 2017; Tumilowicz et al., 2017) and more broadly the critical role of community‐based programs to improve maternal and child health and nutrition (Lewin et al., 2010; Menon et al., 2016; Singh & Sachs, 2013). The Ethiopia MOH Health Extension program and platforms like it provide opportunities to scale‐up MNP delivery but will require strengthening so that frontline workers have the time and ability to effectively support caregivers.

## CONFLICTS OF INTEREST

The authors declare that they have no conflicts of interest.

## CONTRIBUTIONS

AT, TF, JH, FR and LMN designed the evaluation; TF, JH, FR, NA and TTW supervised data collection; FR, TF, JH, NA, AT and HYW conducted descriptive survey data analysis and interpretation which informed the analytical approach of the study presented in this manuscript; AT, JPH, MNNM, TB, RN and GHP designed the study presented in this manuscript; AT, JPH, MNNM, TB and RN conducted data analysis presented in this manuscript; all authors participated in the interpretation of results; AT, JPH, MNNM, TB, GHP and RN wrote the manuscript; all authors read, revised and approved the final version submitted for publication.

## Supporting information

Table S1 Key messages regarding Desta that Health Extension Workers (HEW) and health centre workers were trained to provide to caregivers
Table S2 Factors assessed in analysis based on a priori expectations in facilitating or limiting micronutrient powder intervention outcomes
Table S3 Principal Component Analysis factor loadings and communalities for perception‐of‐use variables rotated using orthogonal varimax rotation
Table S4 Bivariate analysis of the association between various factors and Desta use within last 14 days among all caregivers
