# Supplementary material for: Bottlenecks and predictors of coverage and adherence outcomes for a micronutrient powder program in Ethiopia
Source: Matern Child Nutr. 2019 Oct 17;15(Suppl 5):e12807. doi: 10.1111/mcn.12807 (PMC6856804; doi:10.1111/mcn.12807)
Supplement: Supplementary file 1 — Table S1 Key messages regarding Desta that Health Extension Workers (HEW) and health centre workers were trained to provide to caregivers Table S2 Factors assessed in analysis based on a priori expectations in facilitating or limiting micronutrient powder intervention outcomes Table S3 Principal Component Analysis factor loadings and communalities for perception‐of‐use variables rotated using orthogonal varimax rotation Table S4 Bivariate analysis of the association between various factors and Desta use within last 14 days among all caregivers [file MCN-15-e12807-s001.docx]

**SUPPLEMENTAL TABLE 1** Key messages regarding Desta that Health Extension Workers (HEW) and health centre workers were trained to provide to caregivers

| Topic | Key message |
| --- | --- |
| Composition | - One sachet of Desta provides the recommended daily amount of 15 important vitamins and minerals for young children 6-23 months old. - Desta is not medication. |
| Benefits | - Improves child’s health and development. - Improves child’s appetite. - Improves the nutrition quality of child’s food. - Prevents anaemia and micronutrient deficiencies. |
| Administration regimen | - Give Desta every other day. The child needs to have regular consumption to benefit from Desta. - One box contains 30 sachets. Give all of them to the child within 2 months. Then you will receive a new box until your child reaches the age of 2 years. |
| Preparation | - Wash your and your child’s hands with soap and clean water before cooking and feeding child. - Only add Desta to soft or mashed food. Do not mix Desta with liquids. Examples of the types of foods that could be mixed with Desta include:   - Thick porridge   - Mashed vegetables example potato, carrot, kale   - *Shiro* (stew with chickpea flour-based paste or lentil based thick paste with injera [flat bread with a slightly spongy texture made of *teff*, barley, or maize flour])   - Mashed banana, avocado, mango, or papaya - Do not add Desta during cooking or to hot food. Mix Desta after food has cooled. - Desta should be mixed with a small amount of food that the child can finish during one feeding session. - Pour the whole sachet of Desta into the child’s food and mix well. Content of one whole sachet should be mixed with one meal for one child. It is not recommended to split one sachet into portions given over several meals. - Feed child immediately or within 30 minutes of mixing Desta with food. - Do not share the food mixed with Desta with other household members. - Do not feed more than one sachet of Desta per day. - Store Desta in a clean, cool, and dry place. |
| Side effects | - There are no major side effects associated with Desta. - In the first few days of taking Desta, the child’s stool may be darker than usual. This is completely normal and caused by the iron contained in Desta. Continued use of Desta is advised. - During the first days of taking Desta, the child may have softer stools, mild diarrhoea, or a mild form of constipation, which usually passes in a period of 4-5 days. This does not happen to all children. This is also normal, and it should not be cause for concern. - If diarrhoea is severe, bloody, or with mucous, take the child to the health facility for care as you would have without concurrent use of Desta. |
| Contraindications | - Children suffering from severe acute malnutrition should not receive Desta. Desta is unsuitable for treatment of severe acute malnutrition and children should receive the usual Ready to Use Therapeutic Foods (RUTF) instead. Desta can be used safely after a child is rehabilitated. - Children suffering from malaria should temporarily stop consuming Desta. Once malaria treatment has been completed, children can continue to consume Desta. |

**SUPPLEMENTAL TABLE 2** Factors assessed in analysis based on a priori expectations in facilitating or limiting micronutrient powder intervention outcomes

| ***Socio-demographic*** |
| --- |
| Geographic residence based on strata |
| Socio-economic status (SES) |
| Household Hunger Score (HHS) |
| Caregiver perception of distance to access a HEW is very long |
| Caregiver perception of distance to access health center is very long |
| ***Maternal and child characteristics*** |
| Age of caregiver (years) |
| Caregiver education (years) |
| Age of child (months) |
| Child sex |
| ***IYC feeding practices*** |
| Currently breastfed |
| Child eats from own plate |
| Child ate solid or semi-solid food during 24 hours preceding survey |
| Feeding frequency during 24 hours preceding survey |
| Dietary diversity during 24 hours preceding survey |
| ***Exposure to intervention*** |
| Number of times caregiver met with HEW in last 3 months |
| Number of times caregiver met with health center staff in last 3 months |
| Number of times caregiver met with HEW and health center staff in 3 months |
| Type of nutrition counseling ever received from HEW |
| Type of nutrition counseling ever received from health center staff |
| Type of nutrition counseling ever received from HEW or health center staff |
| Caregiver heard of Desta from HEW or health center staff |
| Caregiver had a Desta child card at time of survey |
| Caregiver ever attended a Desta cooking demonstration |
| ***Perception-of-use factors*** |
| Caregiver perceived increased appetite |
| Caregiver perceived increased activity |
| Caregiver perceived mental development |
| Caregiver perceived increased immunity |
| Caregiver perceived prevention of anemia |
| Caregiver perceived child stronger |
| Caregiver perceived improved child physical growth |
| Caregiver perceived black stool |
| Caregiver perceived loose stool/diarrhea |
| Caregiver perceived constipation |
| Caregiver perceived nausea/vomiting |
| Caregiver reported difficulties in preparing food with Desta |
| Caregiver reported Desta changes taste of the food |
| Caregiver reported forgets to give Desta |
| Caregiver reported reports not knowing enough about Desta |
| Caregiver reported child rejects food with Desta |
| Caregiver reported child doesn’t eat the entire portion |
| Caregiver reported increased appetite is a problem |
| Caregiver reported family not supportive |
| Caregiver reported stockout of Desta at health post |
| Caregiver reported distance/lack of transportation to go to health post |
| Caregiver reported difficulty to meet with HEW |
| Number of perceived positive changes reported |
| Number of perceived negative changes reported |
| Number of reported challenges to give Desta to child |

**SUPPLEMENTAL TABLE 3** Principal Component Analysis factor loadings and communalities for perception-of-use variables rotated using orthogonal varimax rotation

|  |  |  | |  | |  |  |
| --- | --- | --- | --- | --- | --- | --- | --- |
| **Perception variable** | | | **Factor 1 (Perceived positive outcomes)** | | **Factor 2 (Perceived negative effects)** | | **Factor 3 (Perceived challenges to feed Desta)** |
| Caregiver perceived increased appetite | | | **0.21^a^** | | -0.03 | | -0.01 |
| Caregiver perceived increased activity | | | **0.36** | | -0.01 | | 0.10 |
| Caregiver perceived mental development | | | 0.03 | | 0.02 | | -0.02 |
| Caregiver perceived prevention of anemia | | | 0.06 | | -0.02 | | 0.02 |
| Caregiver perceived child stronger | | | **0.39** | | 0.03 | | 0.02 |
| Caregiver perceived increased immunity | | | **0.33** | | -0.01 | | -0.07 |
| Caregiver perceived improved child physical growth | | | **0.45** | | -0.02 | | -0.04 |
| Caregiver perceived black stool | | | 0.11 | | 0.10 | | **0.16** |
| Caregiver perceived loose stool/diarrhea | | | -0.03^b^ | | **0.57** | | -0.08 |
| Caregiver perceived nausea/vomiting | | | 0.05 | | **0.42** | | 0.11 |
| Caregiver reported child rejects food with Desta | | | -0.03 | | -0.04 | | **0.68** |
| Number of perceived positive changes | | | **0.58** | | -0.01 | | -0.02 |
| Number of perceived negative changes | | | 0.00 | | **0.70** | | 0.00 |
| Number of reported challenges to feed Desta | | | 0.02 | | 0.02 | | **0.69** |

^a^ Bold indicated significant loadings >0.15

^b^ Negative loading items indicate more negative responses to the questions were correlated with more positive responses to other items that loaded.

SUPPLEMENTAL TABLE 4 Bivariate analysis of the association between various factors and Desta use within last 14 days among all caregivers

|  | **Caregivers who did not give Desta to child in last 14 days among all caregivers** | | **Caregivers who gave Desta to child in last 14 days among all caregivers** | |  |
| --- | --- | --- | --- | --- | --- |
| **Variable** | N | **Mean / Percentage (95% CI)** | N | **Mean / Percentage (95% CI)** | **P-value** |
| ***Socio-demographic*** |  |  |  |  |  |
| SES score §, mean | 1379 | 0.03 (-0.20, 0.26) | 537 | -0.07 (-0.38, 0.24) | 0.453 |
| **SES quintile:** |  |  |  |  |  |
| Upper, % | 301 | 7.4 (5.7, 9.7) | 82 | 6.1 (3.9, 9.6) | 0.335 |
| Upper middle, % | 284 | 16.0 (12.6, 20.1) | 99 | 14.9 (11.2, 19.6) |  |
| Middle, % | 268 | 25.8 (22.3, 29.7) | 115 | 23.8 (19.5, 28.6) |  |
| Lower middle, % | 257 | 24.6 (21.6, 28.0) | 126 | 29.7 (25.1, 34.6) |  |
| Lower, % | 269 | 26.2 (22.7, 29.9) | 115 | 25.6 (20.9, 30.8) |  |
| Little to no hunger in HH, % | 1363 | 98.9 (98.0, 99.4) | 532 | 98.7 (97.0, 99.5) | 0.730 |
| ***Caregiver*** |  |  |  |  |  |
| Age of caregiver (years), mean | 1379 | 29.3 (28.7, 29.9) | 536 | 28.7 (28.1, 29.4) | 0.159 |
| Caregiver < 5 years education, % | 829 | 67.4 (62.7, 71.9) | 291 | 61.7 (54.8, 68.2) | 0.094 |
| Caregiver highest level of education, %: |  |  |  |  |  |
| None/illiterate | 598 | 49.4 (44.4, 54.4) | 211 | 44.6 (38.0, 51.4) | 0.131 |
| Informal education (read and write) | 71 | 5.6 (4.0, 7.8) | 15 | 3.8 (1.9, 7.4) |  |
| formal education | 710 | 45.0 (40.3, 49.8) | 311 | 51.6 (45.1, 58.0) |  |
| ***Child*** |  |  |  |  |  |
| Age of child (months), mean | 1379 | 14.5 (14.1, 14.9) | 537 | 14.1 (13.5, 14.6) | 0.214 |
| Child sex (female), % | 677 | 49.1 (46.0, 52.2) | 288 | 53.5 (48.5, 58.6) | 0.118 |
| ***IYC feeding*** |  |  |  |  |  |
| Currently breastfed, % | 1182 | 88.5 (86.1, 90.5) | 484 | 89.3 (85.5, 92.3) | 0.627 |
| Child eats from own plate, % | 1211 | 86.0 (83.2, 88.4) | 521 | 96.2 (93.1, 98.0) | <0.001 |
| Child ate solid or semi-solid food during 24 hours preceding survey, % | 1296 | 93.9 (91.9, 95.4) | 528 | 98.2 (96.3, 99.1) | <0.001 |
| Child meal frequency, mean | 1310 | 2.98 (2.85, 3.11) | 531 | 2.96 (2.81, 3.11) | 0.792 |
| Child minimum meal frequency †, % | 899 | 68.2 (63.9, 72.3) | 366 | 68.4 (62.6, 73.6) | 0.965 |
| Child dietary diversity score (CDDS) †, mean | 1379 | 2.02 (1.94, 2.10) | 537 | 2.09 (1.98, 2.20) | 0.204 |
| Child minimum dietary diversity (CDDS>4) †, % | 122 | 5.9 (4.7, 7.5) | 45 | 6.7 (4.3, 10.4) | 0.604 |
| ***Pilot design features*** |  |  |  |  |  |
| Average number of times caregiver met with HEW in last 3 months, mean | 1379 | 1.01 (0.91, 1.11) | 537 | 1.84 (1.70, 1.97) | <0.001 |
| Average number of times caregiver met with health center staff in 3 months, mean | 1379 | 0.56 (0.48, 0.65) | 537 | 0.85 (0.67, 1.03) | 0.001 |
| Average number of times caregiver met with HEW or health center staff in 3 months, mean | 1379 | 1.57 (1.42, 1.72) | 537 | 2.68 (2.46, 2.91) | <0.001 |
| Caregiver perception of distance to access a HEW is very long, % | 150 | 14.0 (9.8, 19.6) | 51 | 11.0 (7.2, 16.4) | 0.197 |
| Caregiver perception of distance to access health center is very long, % | 255 | 24.0 (18.7, 30.1) | 92 | 19.8 (13.9, 27.4) | 0.213 |
| **Type of nutrition counseling ever received from HEW or health center staff:** |  |  |  |  |  |
| Caregivers who received neither feeding nor Desta counseling, % | 310 | 17.9 (15.4, 20.7) | 1 | 0.3 (0, 2.2) | <0.001 |
| Caregivers who received feeding counseling only, % | 414 | 30.1 (26.3, 34.2) | 3 | 0.5 (0.1, 2.0) |  |
| Caregivers who received Desta counseling only, % | 41 | 3.1 (2.0, 4.9) | 9 | 0.7 (0.3, 1.5) |  |
| Caregivers who received both feeding and Desta counseling, % | 614 | 48.9 (44.5, 53.3) | 524 | 98.5 (96.9, 99.3) |  |
| Caregivers who heard of Desta from HEW or health center staff, % | 655 | 52.1 (47.4, 56.7) | 523 | 97.6 (95.8, 98.7) | <0.001 |
| Caregivers who had a Desta child card at time of survey, % | 137 | 10.1 (7.5, 13.5) | 221 | 40.3 (33.9, 47.1) | <0.001 |
| Caregivers who ever attended a Desta cooking demonstration, % | 146 | 24.5 (18.5, 31.7) | 173 | 28.2 (21.9, 35.4) | 0.317 |
| ***Perception-of-use factors*** |  |  |  |  |  |
| Perceived positive outcomes score (factor 1), mean | 556 | -0.37 (-0.54, -0.20) | 537 | 0.39 (0.21, 0.58) | <0.001 |
| Perceived negative side effects score (factor 2), mean | 556 | 0.09 (-0.09, 0.28) | 537 | -0.10 (-0.27, 0.07) | 0.073 |
| Perceived challenges to feed Desta score (factor 3), mean | 556 | 0.14 (-0.05, 0.33) | 537 | -0.15 (-0.28, -0.02) | 0.007 |

§ SES score computed using PCA analysis following DHS methodology (Chasekwa, et al., 2018).

† World Health Organization. (2010). Indicators for assessing infant and young child feeding practices: part 2: measurement.
